# Follow #eHealth2011: Measuring the Role and Effectiveness of Online and Social Media in Increasing the Outreach of a Scientific Conference

**DOI:** 10.2196/jmir.4480

**Published:** 2016-07-19

**Authors:** Marcel Winandy, Patty Kostkova, Ed de Quincey, Connie St Louis, Martin Szomszor

**Affiliations:** ^1^ Horst Goertz Institute for IT-Security Bochum Germany; ^2^ University College London London United Kingdom; ^3^ Keele University School of Computing and Mathematics Keele United Kingdom; ^4^ City University London United Kingdom; ^5^ Data Science London United Kingdom

**Keywords:** social media, social media networks, Web conferencing, marketing

## Abstract

**Background:**

Social media promotion is increasingly adopted by organizers of industry and academic events; however, the success of social media strategies is rarely questioned or the real impact scientifically analyzed.

**Objective:**

We propose a framework that defines and analyses the impact, outreach, and effectiveness of social media for event promotion and research dissemination to participants of a scientific event as well as to the virtual audience through the Web.

**Methods:**

Online communication channels Twitter, Facebook, Flickr, and a Liveblog were trialed and their impact measured on outreach during five phases of an eHealth conference: the setup, active and last-minute promotion phases before the conference, the actual event, and after the conference.

**Results:**

Planned outreach through online channels and social media before and during the event reached an audience several magnitudes larger in size than would have been possible using traditional means. In the particular case of eHealth 2011, the outreach using traditional means would have been 74 attendees plus 23 extra as sold proceedings and the number of downloaded articles from the online proceedings (4107 until October 2013). The audience for the conference reached via online channels and social media was estimated at more than 5300 in total during the event. The role of Twitter for promotion before the event was complemented by an increased usage of the website and Facebook during the event followed by a sharp increase of views of posters on Flickr after the event.

**Conclusions:**

Although our case study is focused on a particular audience around eHealth 2011, our framework provides a template for redefining “audience” and outreach of events, merging traditional physical and virtual communities and providing an outline on how these could be successfully reached in clearly defined event phases.

## Introduction

Measuring the impact or influence of a particular scientific or business event is an important part of evaluating its success and the effectiveness of its promotion. Although social media promotion is a “must” for most commercial and academic events, little interest has been given to defining new audiences participating virtually and physically as well as analyzing the impact and outreach of all individual social media channels used in promotion and scientific outreach. Traditionally, the impact of a scientific conference has been measured by the number of attendees and the number and quality of publications (in terms of acceptance rate and citations). These measures are based on the traditional means of communication with physical communities: face-to-face meetings and printed media. However, in an increasingly widely connected world, the use of social media and novel online channels spanning the traditional physical and virtual divide has revolutionized communication outreach, community engagement, and the overall impact of a scientific conference that can embrace and utilize the new media channels.

In this context, it is important to know the role and effectiveness of online and social media channels in engaging a community. There is a vast amount of research around the usage and effects of social media (eg, [1-4]). Among the factors that most research focuses on are analyzing the dynamics of social networks, information diffusion and propagation, users influence, and attention. However, little attention has been given to investigating the impact of a social network on a physical community, around a single topic, over an extended period of time, and how intensive, face-to-face interaction and virtual socio-patterns at a conference affect the size and constituency of the virtual network. Secondly, most research has looked into an isolated social network or media, such as Twitter or Facebook, but investigating the role of these channels in creating and engaging a community has not been addressed. Finally, most existing research investigates a snapshot image of the entire social network (one-way mining data from the social network, eg, Twitter). In contrast, we have conducted a longitudinal study over 6 months (two-way sending data to and mining from social networks).

In this paper, we make the following contributions:

We define a framework of (1) media channels and their impact factors as well as (2) establishment of longitudinal phases and analysis measures. With the first part of the framework, we aim to study the relationship between an online and real community, and we compare traditional and new impact factors of the outreach of a real-world scientific event. The second part of the framework aims to analyze how successful each media channel is throughout different phases of planning and running an event.

We evaluate our framework in a case study of a real event (ie, the eHealth 2011 conference), which took place in 2011 in Malaga, Spain. We present a detailed analysis of the data we collected through a longitudinal evaluation over different phases to determine the outreach of each media channel and how to calculate the detailed activity on creating and engaging an online community around a conference.

Finally, we discuss the results of our case study and aim to answer the question of which role is best suited for each media channel before, during, and after a conference.

The objective of our research is to investigate methods to determine the impact of different media channels on a real event over traditional research event dissemination methods. To this end, we define and suggest a strategy for promotional phases before, during, and after the event, and an outreach score as a measure to determine the impact.

### Related Work

The availability of Twitter datasets has created a rapid increase in research projects across a range of domains investigating influence, propagation, information diffusion, and social network topology. There has been some interest in investigating the role of social media to improve user experience and engagement with conferences, for example, mobile phone apps such as Conference Navigator presented at UMAP 2011 [5] and conference organizing apps using social media [6]. However, neither work addresses the use of multiple channels of outreach beyond the event itself.

Research on the dynamics and influence of the network itself primarily address various issues of creating influence and activity versus passivity of users to post, retweet, and mention. Influence and passivity scores investigated by Romero et al [2], Meeder et al [7], and Bruns et al [8] introduced methods to retrospectively determine follower growth on Twitter accounts. In contrast, we collect the data on the number of followers of our account on a regular basis and we also observe the numbers of users who unfollow. Unlike most studies that investigate a snapshot of the social network, our research looks into a longitudinal community behavior and long-term impact. Golbeck [9] conducted a longitudinal study of membership growth in various social networks and observed a linear increase in most cases. However, her research looks into general growth of an entire social media network. Instead, our research targets a specific community across various media around a single event. Russo et al [3] presented a longitudinal study investigating a relationship between tagging and attention on a variety of social networks. Cosley and Lan [4] studied social influence using Wikipedia. Although these works demonstrate that people with a high density of interconnection actually share less information about the content and context.

An attempt to investigate the correlation between social networks and real networks was investigated by Tugkeci [10], who looked at 617 users using qualitative and quantitative methods. This study revealed that there was no difference in the number of offline friends between those who made new friends online and those who did not. However, the aim of our study is to investigate a real community of professionals with interest in eHealth who had an opportunity to meet face-to-face at the conference. Thus, the online network converged to a face-to-face interaction.

Research into dynamics and activity of user influence on Twitter has also been flourishing. For example, Cha et al [1] analyzed across three measures: indegree (ie, number of followers), retweets, and mentions. They analyzed a large dataset over (almost all) Twitter users to investigate the influence of single user types and how this influence can remain constant across different topics and over time. Although we also use indegree, retweets, and mentions among our measures for outreach, we do not aim to investigate a single influence but a community as a whole, and we aim for high-density spider networks around a single topic. Although Cha et al and others analyze only a snapshot of activity of all users at the time of their data crawling, we analyze the temporal change of the users’ network around an event.

Finally, there is also research focusing on social media usage in academic conferences. For example, the study by McKendrick et al [11] demonstrated the use of social media at health care conferences. They analyzed and categorized tweets that were posted before, during, and after the event. Wen et al [12] analyzed the usage of Twitter for several academic conferences over a time period of 5 years. Although both works show new insights into the usage and network structures of Twitter around conferences and their change over time. Moreover, Wen et al focus on datasets 2 weeks around each event, whereas we not only define larger longitudinal phases that range from several months before the conference up to weeks afterwards, but we also generate data by ourselves, turning the conference audience into a real-world laboratory. Other related areas of research include analysis of why and how people in particular use Twitter during academic conferences [13-18]. A different research direction—measuring interaction socio-patterns and close proximity interaction at a scientific conference using radio-frequency identification (RFID) sensors—was conducted by Barrat et al [19] and Szomszor et al [20], but these studies focused on the physical interaction during the event rather than the wider outreach of scientific outputs. In contrast, we aim to look at what social media channels are best suited to increase outreach and when. Being more closely aligned to our goals, a few case studies [21] aim to examine how to use various social media to increase outreach of scientific conferences. But, most of these are oversimplified and concentrate on one particular channel. Instead, we provide a framework of how to integrate several channels with their specific roles in longitudinal phases and to measure their outreach in terms of certain impact factors. This is achieved by addressing the following questions:

1. What is the relationship between an online and real community in terms of coverage and overlap and what are the impact factors of different (social) media channels through which the communities are built?

2. How successful is each social media channel in the phases of planning and running a real event (ie, a conference)?

3. What is the overall outreach and how to calculate the detailed activity in creating and engaging an online community around a conference?

## Methods

### Framework

In our framework, we considered the following online media channels and their role in establishing extended outreach and community growth:

1. Twitter to provide general, dynamic information about the conference, promote the event, link to relevant news and other information, define a dedicated hashtag for the conference, and actively establish a community of potentially interested followers;

2. Facebook to provide general, dynamic information about the conference, promote the event, link to relevant news and other information, and connect to the Twitter account;

3. Flickr to create a dedicated group for the conference and post images and abstracts of all posters of the conference;

4. Liveblog to provide live blog messages during the event and link in all tweets from Twitter that used the conference hashtag;

5. Website to provide general (static) information about the conference and provide links to the submission system and to all other media channels; and

6. email to send call-for-papers and call-for-participations to mailing lists.

Each of these channels has a different impact on the outreach of the conference. Among the “traditional” impact measures are the number of conference attendees, the number of printed proceedings that are sold, and the number of papers downloaded as electronic versions. These traditional impact measures are often related to the physical community of the conference and the associated traditional media channels. In contrast, social media channels offer a new set of impact measures. These include the number of followers of a social media account, the number of tweets or posts that are related to the conference, and (compared to downloads of papers) the number of page visits on the Flickr group that hosts the images and abstracts of the posters presented at the conference. Multimedia Appendix 1 summarizes the impact measures that we determined based on the corresponding media channels.

#### Establishment and Measurement

To measure the outreach of the event with the new impact measures, we first set up a number of media channels for the event. Next, we defined five phases along the timeline of the event, ranging from early time before the event up to a time after the event. As such, we could analyze the growth and change of the virtual and physical communities around this real event in a longitudinal manner.

#### Longitudinal Phases

The five longitudinal phases were oriented around the Fourth Institute for Computer Sciences, Social Informatics and Telecommunications Engineering (ICST) International Conference on eHealth (eHealth 2011), which took place in Malaga, Spain, on November 21 to 23, 2011. Instead of simply looking at the timelines before, during, and after the event, we differentiated the beginning of the time before, usually used for setting up things, and the very last part of the time before (“last minute”), when usually the latest news are announced and advertisements are made to give a final push in attracting attendees. The resulting phases were:

1. Setup phase (May 10-27): setup of social media accounts, website, email list, etc;

2. Active promotion phase (June 1-November 2): community growing phase before event;

3. Last-minute promotion phase (November 13-17): announcing latest information about the event a few days before;

4. Actual event phase (November 21-23): activity during the conference; and

5. Postevent phase (November 24-December 5): community behavior after the event.

6. For each phase, we measured the activity on each channel and aimed to determine its impact on the community outreach. Moreover, we measured, but we also actively generated data on those channels. This is different compared with most prior research; essentially, we let the conference become a real-world laboratory where we not only analyzed the data, but also performed research on the response to our actions. By having different phases, we could also look at which channel performed better in which phase. Naturally, the different spaces of time around an event (before, during, after a conference) will have different activities involved (eg, announcing a conference after the event has passed is quite useless). Based on the five phases and the measurements taken during these phases, we aimed to determine which channel was best suited for which phase.

#### Procedure

Firstly, we set up the media channels as listed in Multimedia Appendix 2. The Facebook page was created to promote the event conference (eg, place and date of the conference, submission deadlines) and to provide information about the conference such as changing dates, announcing keynotes, or links to subpages of the website later on.

The Twitter account @eHealthConf was then linked to the Facebook page so that messages posted on the Facebook page were automatically posted to the Twitter account, including a link to Facebook if the message was longer than 140 characters (the limit of tweets on Twitter). To increase the number of followers of @eHealthConf, Twitter accounts of similar events known to the organizers were identified and followed along with relevant eHealth organizations, research groups, and researchers. Twitter users who followed those accounts were then also identified and followed (similar to snowball or chain sampling).

The official hashtag of the conference of #eHealth2011 was decided on and publicized via Twitter, Facebook, the conference website, and at the event itself during the welcome session. Our Liveblog system also included all tweets and retweets of the @eHealthConf account and tweets using the hashtag #eHealth2011. For the poster session at eHealth 2011, we setup a Flickr gallery where the poster presenters could upload their posters for public viewing, which was promoted during the poster session itself, on Twitter and Facebook, and also on the conference website.

All channels were linked to from the conference website and verbally promoted during the introductory session at the conference itself.

#### Measurements

For each media channel, we defined a set of measurements for the five longitudinal phases. We took the measurements (when possible) on a daily basis (ie, summarized the value of a measurable element at the end of a day).

For Twitter, we used the following measures on a daily basis:

1. Followers: number of users following the event account (measured via the Twitter email notification on new followers).

2. Followers lost: number of users who stopped following the event account (measured via the third-party service TwUnfollow [22]).

3. Retweets: number of retweets of the event account (measured via the Twitter email notifications); this pertained only to those retweets that used the official “retweet” application programming interface (API) of Twitter. Other ways of retweeting (eg, manually writing “RT...<account name> <message>”) were counted with mentions.

4. Mentions: number of tweets from other users that contained the event account name (measured via the Twitter email notifications about mentions). This did not include retweets that were done via the official retweet API of Twitter (although some clients show this as a retweet in the timeline).

5. Users receiving retweets: number of users to which messages from the event account were retweeted (via the official retweet API only). The data were derived from the Twitter email notifications (which contained information such as “@XYZ retweeted to N followers...”). We took the sum of these numbers per day, whereas when retweets of the same user occurred, we counted only once and used the maximum number (due to changes in followers of that user during the day). We did not subtract duplicates here (eg, users that received the same retweet from two or more followers of our account).

To analyze the outreach of the Facebook page, we took three measures because they are provided by the weekly Facebook status update (via email notification):

1. Likes: the number of Facebook users that liked our Facebook page;

2. Posts: the number of posts or comments on the page’s wall, either made by ourselves (the page) or by others; and

3. Visits: the number of visits of the Facebook page.

On Flickr, the number of page views was the relevant measure. This could be on individual posters (or photos) or for the gallery front page as a whole.

For the Liveblog, we measured the number of online users that were connected to the service at a given time. We differentiated between the highest number of participants at any one time (for calculating maximum) and the number of total online users on a particular day. This measurement was only taken during the event because the Liveblog was only available in this phase.

The measure for the email lists was the number of email recipients. This was slightly different from the number of registered email addresses in the list because some emails could be bounced due to various reasons (eg, address not valid or someone had set up a notification of absence).

The typical measurement of a website is the number of (daily) page visits. In addition, geographical information about the visitors could be of interest.

Finally, for the attendees, we counted the number of persons who were physically present at the conference.

#### Outreach Score

Based on the continuously taken measurements, we calculated the outreach of each media channel for each longitudinal phase. The idea was to compare the different media channels and their outreach performance to identify the best-suited channel (or channel mix) for each phase. Previous work into promotion of scientific online content using various channels by de Quincey et al [23] was a step in the right direction, although not linked to a physical event. Therefore, we calculated three values: maximum outreach, mean outreach, and total outreach for each phase. Maximum outreach shows for each channel the maximum number of users we could reach on a single day during a particular phase. This did not necessarily mean we actually reached them because they could have missed or discarded the message. However, it was an indicator of the maximum size of a virtual community. Mean outreach for each channel was the arithmetic mean of the number of users that we reached daily during a particular phase. Again, this did not necessarily mean they actually read a message or were actively involved. However, it was an indicator of the community growth when we looked at this measure over time. Total outreach for each channel summarized the number of users that we reached in a time period of a particular phase. Although we cannot completely rule out duplicates (eg, access to the website on two different days could have originated from the same or from different users), the total outreach was an indicator how many users could be reached in total during a given phase.

We chose these outreach scores because we could not measure the exact numbers due to overlaps. Although for certain media (eg, Twitter) it was possible to rule out overlaps by using intensive data crawlers over time (capturing and analyzing the links of followers and subtracting duplicate users), we did not use this in the first place. Moreover, for some media channels, it was more or less impossible to rule out duplicate users (eg, page views on a website from the same IP address). However, we attempted to cleanse the data to reduce the potential influence of duplicates (eg, we did not summarize the number of attendees for all days of the event when they were obviously the same because we know from the registration list).

Table 1 shows the resulting outreach scores of our framework for all channels and phases. Note that all outreach scores were defined within a phase and did not include the data of the other phases. Some scores contained adjustments to reduce effects of duplication. For example, for the total outreach of Twitter during a phase, we did not summarize all followers because this would most likely include too many duplicates. Instead, we took the number of followers at the end of a phase, added the sum of the followers that we lost during this phase, and added the maximum of users receiving retweets for this phase. The latter (adding the maximum instead of the sum) is an adjustment we made because we did not know the number of followers lost of those users receiving retweets. Where we could clearly identify the users (email list and attendees of the conference), we counted the real persons as the total outreach.

**Table 1:** Outreach scores of our framework.

Channel	Maximum outreach	Mean outreach	Total outreach
Twitter	Maximum(followers + users receiving retweets)	SUM(followers + users receiving retweets) / COUNT(followers + users receiving retweets)	SUM(followers at end of phase) + SUM(followers lost) + maximum(users receiving retweets)
Facebook	Maximum(likes + visits)	SUM(likes + visits) / COUNT(likes + visits)	SUM(likes + visits)
Flickr	Maximum(views)	SUM(views) / COUNT(views)	SUM(views)
Liveblog	Maximum(online users at the same time)	SUM(online users) / COUNT(online users)	SUM(online users)
Email	Maximum(recipients)	SUM(recipients) / COUNT(recipients)	COUNT(recipients)
Website	Maximum(visits)	SUM(visits) / COUNT(visits)	SUM(visits)
Attendees	Maximum(persons)	SUM(persons) / COUNT(days of event)	COUNT(persons)

The reason we looked at three values (maximum, mean, and total) was that they showed a different view of community growth and interconnectivity. For example, a maximum may be very high during a phase, but this could be the result of only a single action. The mean, however, could show the density of interactions during a time period. The total shows the effectiveness over the whole period of a phase.

The essential idea of our framework was to take the outreach scores as previously defined and evaluate them for each phase. This meant these figures were repeated for each of the five phases to receive the overall view. For better comparison, the length of each phase needed to be normalized.

## Results

Table 2 details the outreach scores during each phase and compares the different media channels.

**Table 2:** Comparison of outreach results for each phase.

Phase		Setup			Active promotion			Last-minute promotion			Actual event			Postevent		
		Max	Mean (SD)	Total	Max	Mean (SD)	Total	Max	Mean (SD)	Total	Max	Mean (SD)	Total	Max	Mean (SD)	Total
**Channel**																
	Twitter	19	13 (4)	20	2343	555 (339)	3100	1011	721 (155)	1023	1562	1236 (434)	1569	665	605 (19)	682
	Facebook	—	—	—	105	75 (16)	1129	89	89	89	181	181	181	112	112	112
	Flickr	—	—	—	—	—	—	—	—	—	—	—	520	833	165	1489
	Liveblog	—	—	—	—	—	—	—	—	—	20	139 (50)	416	—	—	—
	Email	1989	1431	1989	2046	2018	2046	2047	2047	2047	2047	2047	2047	—	—	—
	Website	72	32 (20)	870	164	45 (25)	7342	181	102 (44.9)	817	307	194 (109)	583	81	46 (21)	555
**Traditional impact measure**																
	Attendees	—	—	—	—	—	—	—	—	—	74	74	74	—	—	—
	Proceedings	—	—	—	—	—	—	—	—	—	—	—	—	—	—	4130
	Sum	2080	1476	2879	4747	2693	13617	3328	2959	3976	4191	3869	5390	1691	929	6968

The total outreach during the event was 5390. The maximum outreach on a single day during the event was over 4191 because this did not yet include the traditional outreach of proceedings. Note that these calculations could be even higher, in particular in the other phases, because some measurements were not or could not be taken. Also, the number of proceedings (23 sold books and 4107 downloaded online articles) occurred later than our defined postevent phase, but for completeness, we added them to the total outreach of the last phase. Finally, the resulting numbers did not eliminate duplicates. For example, a physical attendee could visit the website, retweet a message from the conference Twitter account, and post something in the Liveblog while visiting the Facebook page. Hence, this is an upper bound of the outreach.

**Figure 1:**
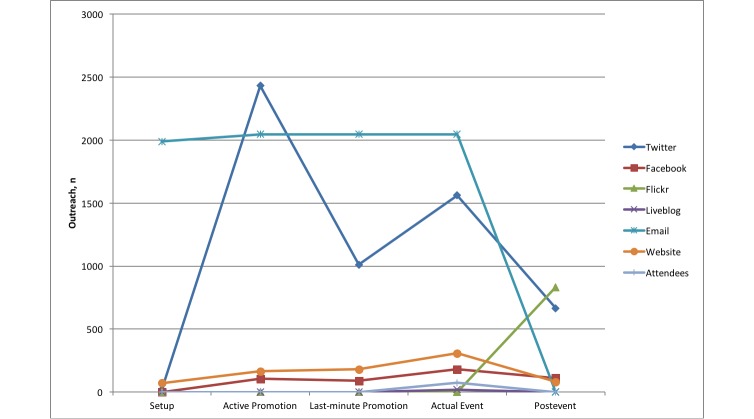
Comparison of maximum outreach of all channels.

**Figure 2:**
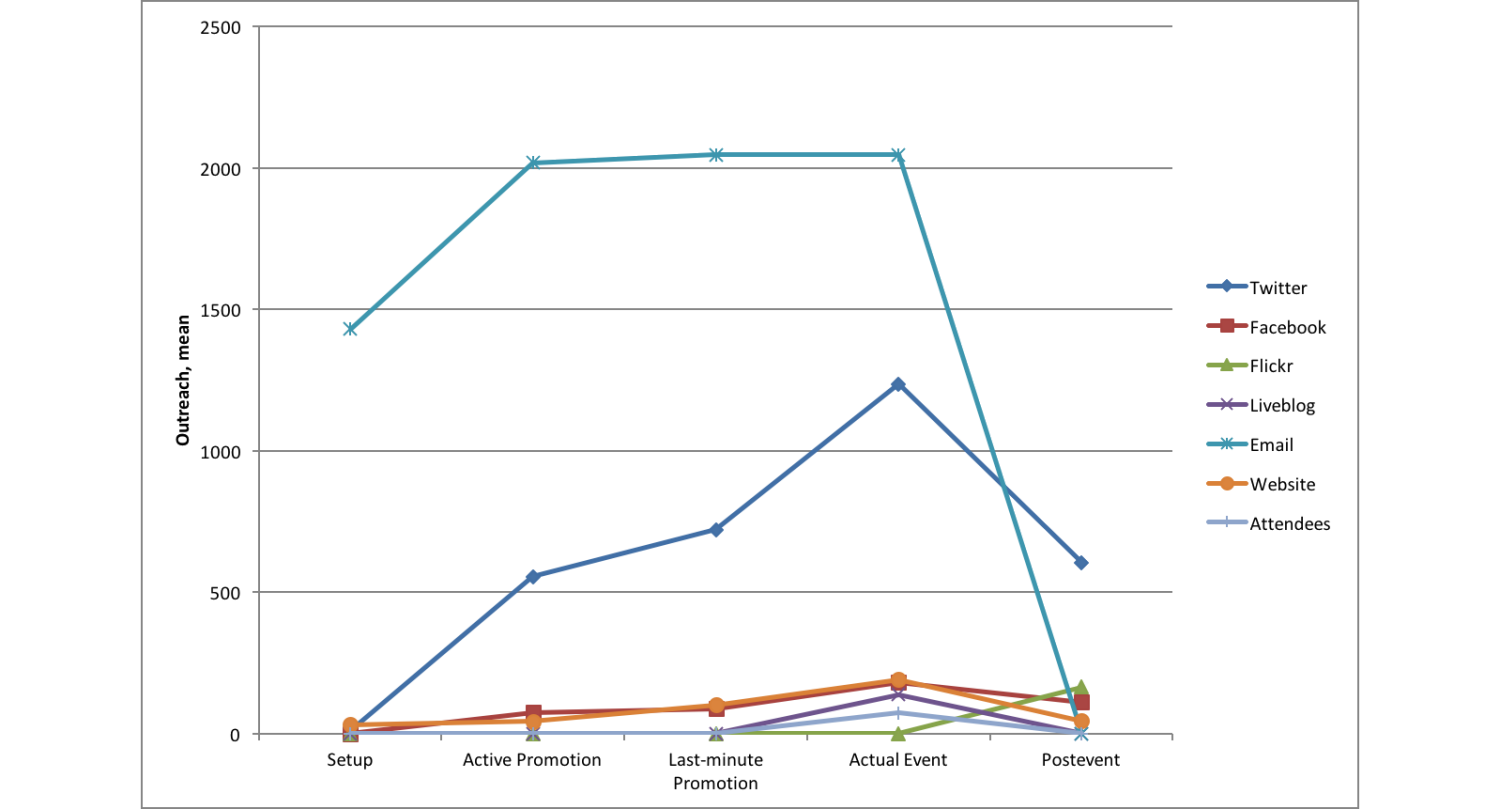
Comparison of mean outreach of all channels.

**Figure 3:**
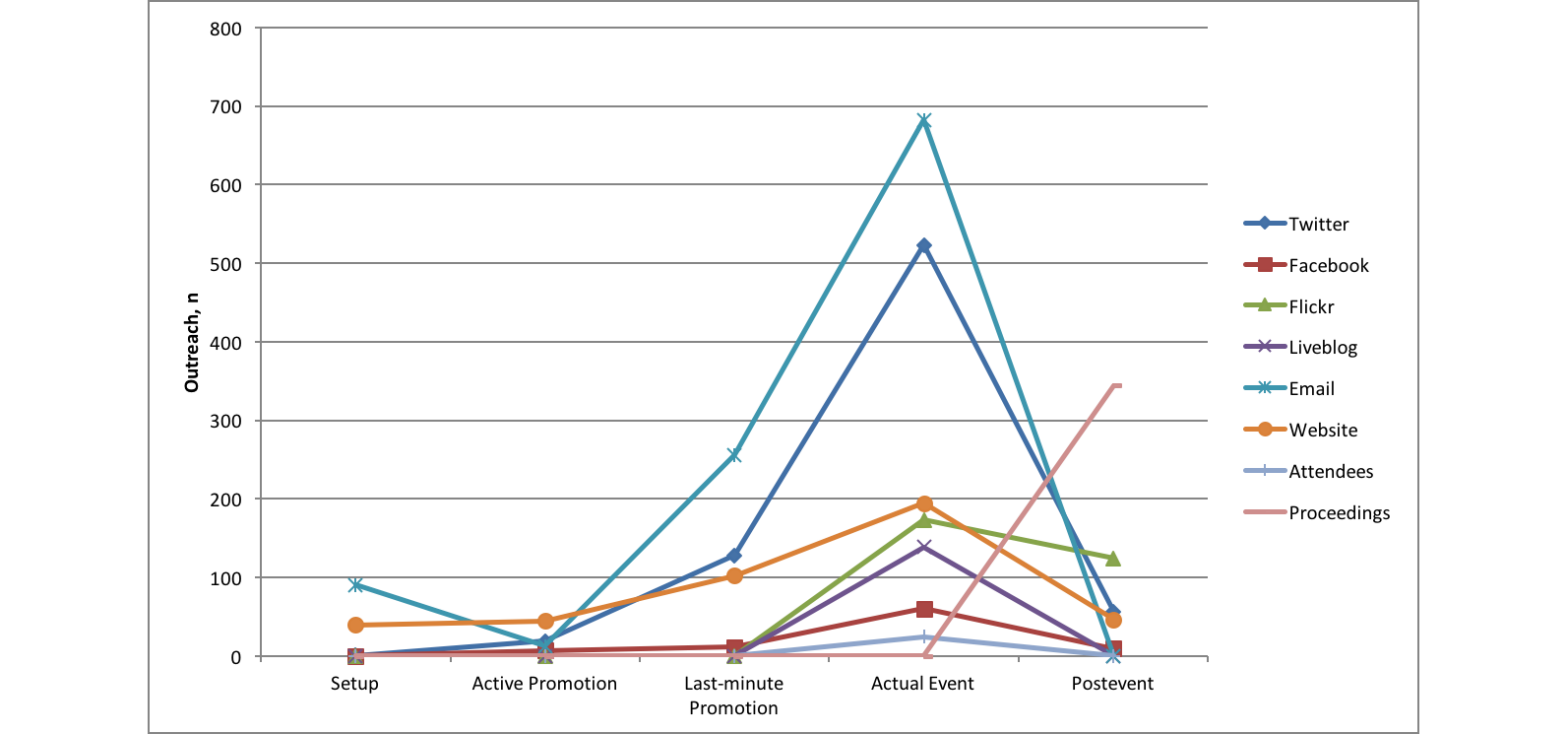
Comparison of total outreach of all channels (normalized to length of each phase).

Comparing the outreach results of the different channels over the five phases, we can identify certain differences. Some channels seem to be more effective in certain phases than in others. Figures 1 to 3 show comparative diagrams for the outreach scores maximum outreach, mean outreach, and total outreach, respectively. Total outreach was normalized to the length of each phase in days.

In the first phase (setup), we had very low outreach scores in most cases because the channels had just been set up. The numbers of page visits, Twitter followers, etc, were not expected to be as large right from the beginning. One exception was the email list, which was set up very quickly (based on existing lists of recipients from previous conferences) so that a first call-for-papers could be sent out to a large number of people early. This is the traditional way of announcing a conference, in companionship with establishing a conference website.

For the second phase (active promotion), however, Twitter and email, in particular, had much more outreach than the other channels in terms of maximum and mean. If we look at the total outreach instead (see Figure 3), the traditional website has accumulated the most outreach over the period of this phase. Interestingly, this is contrary to the much higher peaks for email and Twitter in maximum and mean outreach. An explanation for this can be that Twitter is a more dynamic medium with respect to retweets and mentions, whereas website visits are rather a “static” but continuously performing outreach.

The third phase (last-minute promotion) differed a little from the previous one, although it was also related to promotional activities. Email and Twitter were still the most dominant channels in maximum and mean outreach. For the normalized total, however, Twitter overtook the website. This could be explained with the quickly increasing number of followers in this phase.

In the fourth phase (actual event), the previously “less important” channels gained more significance. The Liveblog and the attendees were present only in this phase; therefore, they had their (only) peaks here. In addition, the maximum and mean outreach of Facebook and the website also had their highest peaks here. However, email and Twitter were still the best performing channels, this time also for the (normalized) total outreach. Although one would expect those channels that offered highly dynamic interactions (eg, mentions/retweets in Twitter, likes/posts/comments in Facebook) to be the ones that outperformed all other channels, we saw that this was not true for Facebook in our case study. Liveblog, Flickr, and the website were better than Facebook in the normalized total outreach. The website channel was even better than Facebook in maximum outreach.

The last phase (postevent) showed a very different result. The outreach of Twitter declined (and email because no emails were sent after the event, of course). In particular, Flickr had a high outreach in all three categories (maximum, mean, and total). But, because this phase was kind of a wind down phase, it was clear that channels with a more archive-like character were more effective in this phase. This was particularly true for proceedings (which are usually read by an increasing number of people after the conference) and Flickr (as a new medium to show the conference posters to a wider community).

Twitter was far more effective than Facebook as a social media channel for a scientific conference such as in our case study. Twitter and email were the most effective channels during all phases up to the actual event. During the event, channels such as the website (eg, showing information about the program) could be enhanced with media channels that allow active participation (eg, Twitter, Facebook, and Liveblog). Interestingly, our case study showed that Facebook had less relevance, whereas the Liveblog seemed to be a good addition to support active discussion and allow people to remotely participate at the conference. Using our novel methods, the Liveblog engaged 5.6 times more “virtual participants” than those physically attending. After the event, traditional media such as proceedings (online and offline) can be enhanced with special-purpose social media such as Flickr to increase the outreach of presentations. In particular, Flickr exposure of posters gave access to seven times more users during the conference and overall, including the postconference phase, 20 times more than those who would have seen them physically. Overall, phase 2 saw the highest total (it was also longest phase), but the highest mean outreach was during the conference itself (3869 in phase 4 driven predominately by Twitter).

Although the results are only from one event (and with a relatively focused target group), they are useful as recommendations to structure and plan media channels for other (scientific) conferences. Similar to the body of socio-patterns research, the generalizability of results from various experiments with real-world participants is a challenge [24]. However, the setting of our case study around a conference makes it a standard scenario. The framework itself is generalizable and could be adapted to other events to include other media channels.

### Analysis of Data per Channel

In this section, we present and analyze the detailed data of the different media channels we used to promote the real event. In particular, we look in more detail at the data and results on a per-channel basis.

#### Twitter

##### Followers

**Figure 4:**
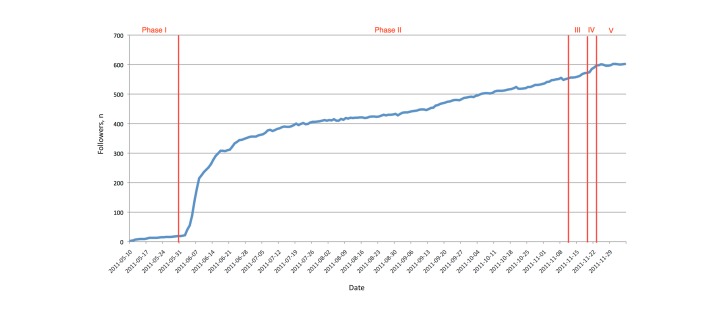
Twitter: number of followers through the different phases. Phase I: set up; phase II: active promotion; phase III: last-minute promotion; phase IV: actual event; phase V: postevent.

Figure 4 shows the number of followers measured over time. The data were taken from email notifications from Twitter about new followers subtracted by the people who unfollowed as reported by the service “TwUnfollow” [22].

We observed that the five phases of the promotion timeline could be matched to five data periods in the graph. The first period matches with the setup phase, which had unsurprisingly low followers because the Twitter account was new and known only to the organizers themselves at this stage. The second period (active promotion phase) started with a rapid increase in the number of followers within a few days. This rapid growth matched with the time (approximately a week) when we started to follow other people (up to a maximum of 2000, a limit set by Twitter at that time to avoid spam accounts). This rapid growth was followed by moderate but continuous growth for the rest of the second phase, the active promotion phase. In the third period (last-minute promotion phase), we sent promotional and announcement tweets about the program and invited speakers. During the conference, there was a smaller increase, probably resulting from an increased number of tweets in the actual event phase. Finally, after the conference, the number of followers remained more or less constant.

During the short period in the beginning of the active promotion phase when we increased the users that we followed (up to 2000), a number of people followed us back immediately. However, most users did not follow us back. For example, on June 6, 2011, we followed 1579 users and 1434 did not follow us back. As our number of followers increased during the third period, we had a fairly constant ratio of followers versus following; at the end of this period (at the time of the conference), the number of users not following back was 1587, whereas we followed 1998 users. We measured the ratio over two months (September and October 2011) and during the time of the conference, but it kept almost constant. Therefore, we had a fairly constant number of people following us back (approximately 400). Some immediately followed back when we followed them, so we can only speculate about their interest in our account. We can assume some of them only followed because they were followed.

In addition, our final number of followers (more than 600) meant that we could attract approximately 200 users to follow our account without following them. We can assume they were directly interested in our account (ie, the conference and the tweets about it).

##### Followers Lost

**Figure 5:**
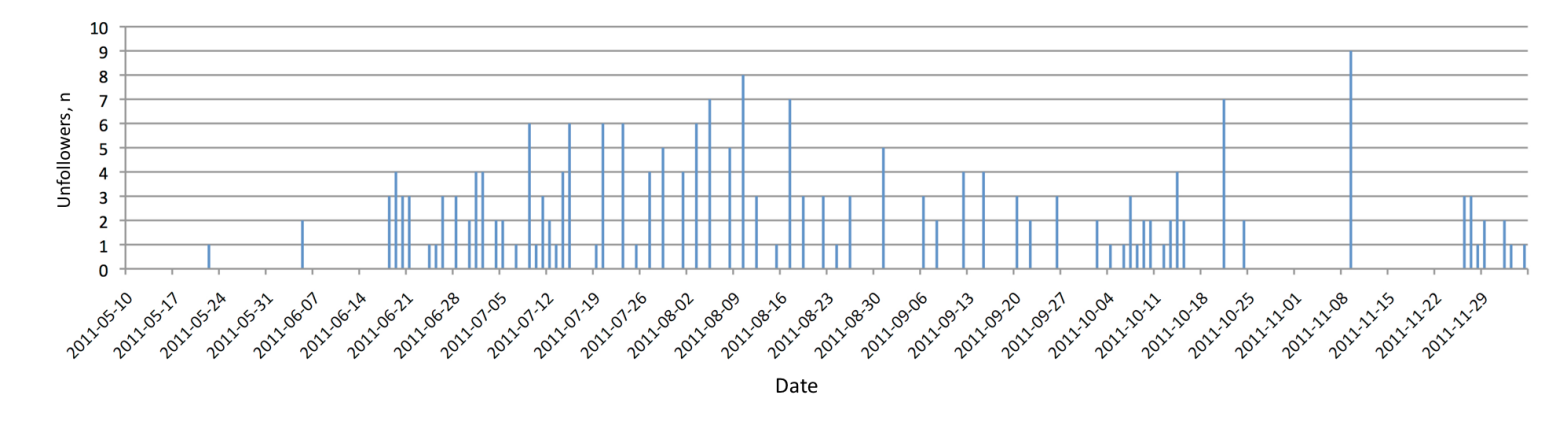
Twitter: number of followers lost.

The number of followers we lost, as reported by the service TwUnfollow, was at a relatively low rate throughout the overall time period (see Figure 5). Although the timeline included a few peaks, as Figure 5 shows, we could not accurately assign the “unfollowers” to specific dates. The TwUnfollow service sometimes aggregated the followers lost for a few days. We assigned these numbers to the day reported by TwUnfollow. The website of TwUnfollow itself stated that due to high load “it may take up to 48 hours until unfollows appear in your history.” Hence, these reports are only an approximation of specific days. Unfortunately, Twitter does not provide a comprehensive interface to analyze unfollowers.

##### Tweets and Retweets

**Figure 6:**
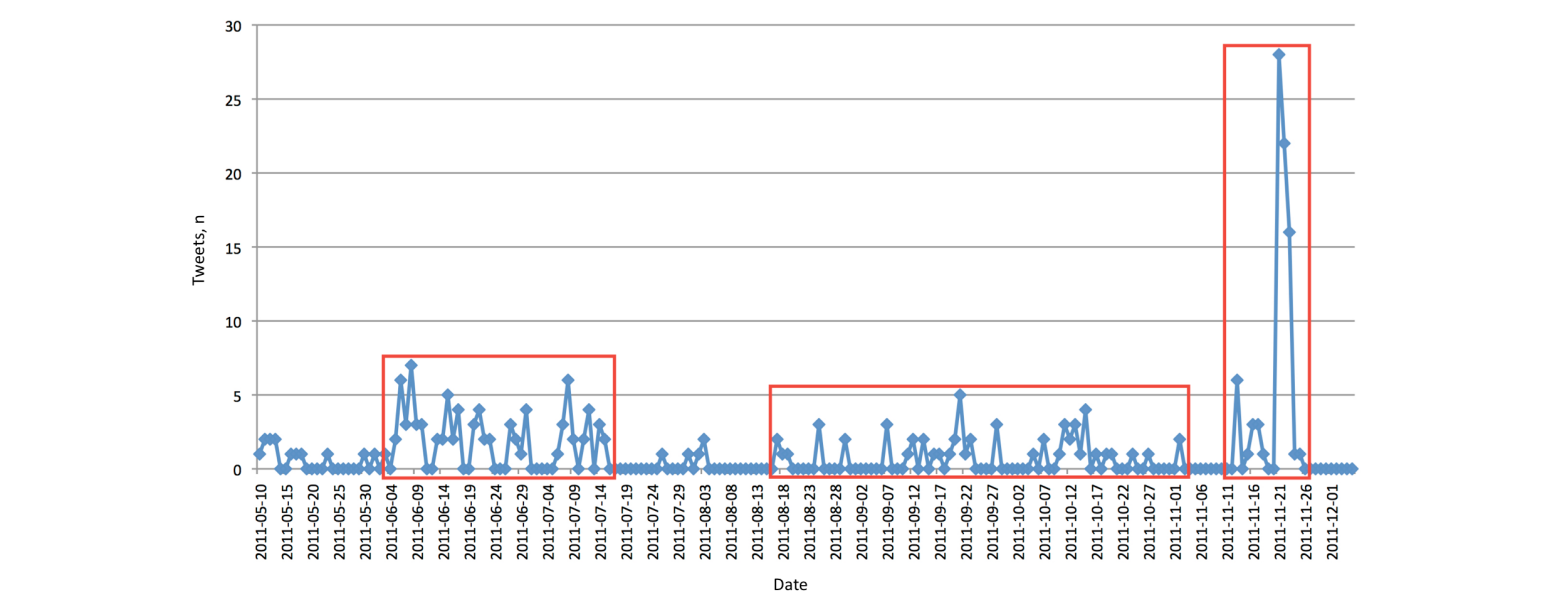
Twitter: number of tweets. The three major periods of activity (in red boxes) correspond to when initial announcements about conference were made, when deadline extensions and reminders to register were made, and during the conference itself, respectively.

Figure 6 shows the number of tweets that we sent through the conference Twitter account. Our tweet activity was a result of the different phases of promotion as described previously. There were three major periods of activities in tweets: (1) in June 2011, when we made initial announcements of the conference (eg, posting the call-for-papers); (2) from August to October 2011, when we announced deadline extensions and reminders to register; and (3) November 21 to 23, 2011, during the conference itself.

The third period had the highest volume of activity because information was posted about ongoing talks and other information during the conference. The first and second periods belonged to the active promotion phase and showed that we had more activity in the first quarter and second half of this phase. However, as the number of followers before showed, there was a steady increase, even in times when we had low activity in tweets.

**Figure 7:**
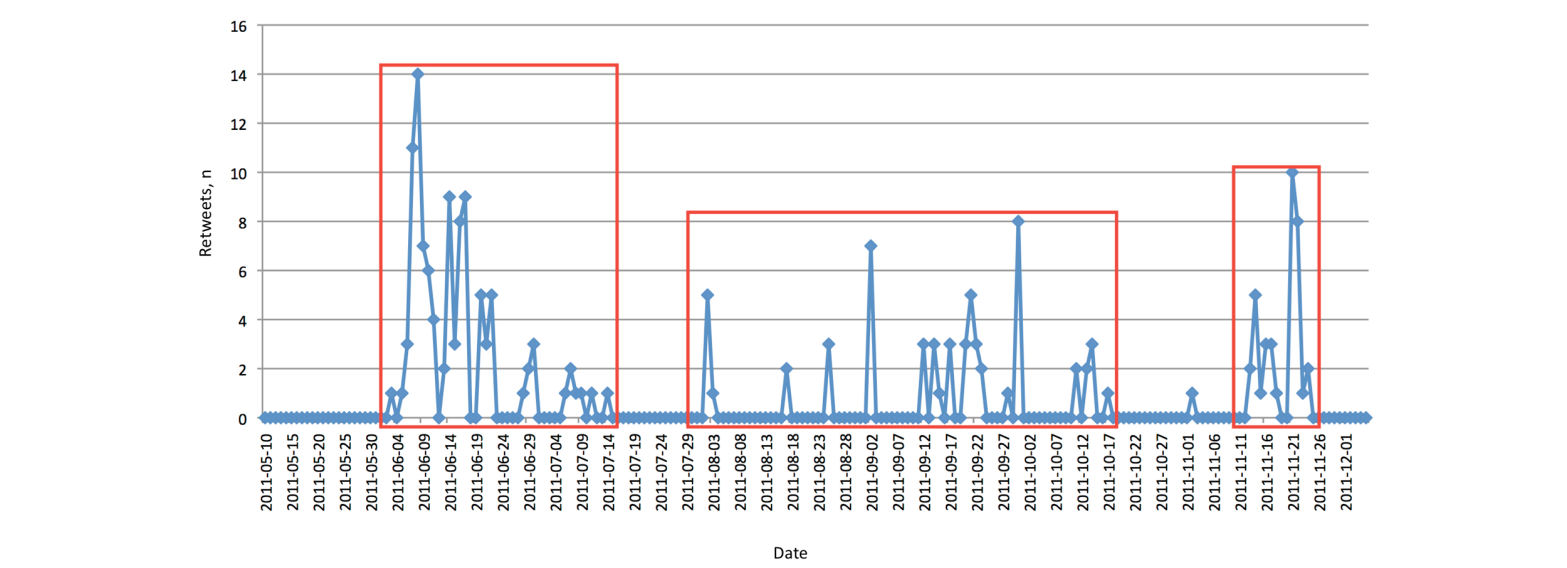
Twitter: number of retweets. The three major periods of activity (in red boxes) correspond to peaks in tweet activity.

In addition to the tweets, we also analyzed the corresponding retweets (see Figure 7). As expected, there was a peak of received retweets during the conference (November 21-23, 2011). However, we also had a number of higher peaks before which matched the three periods of our tweet activity (of course, there would no retweets by other users to be expected if we have no tweets).

##### Mentions

**Figure 8:**
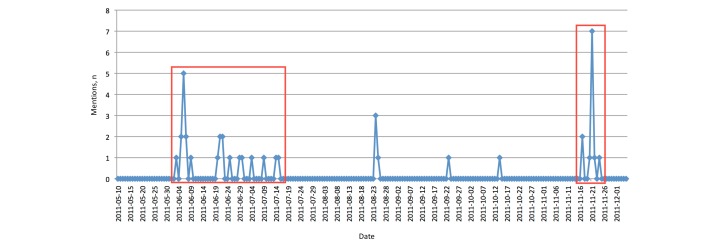
Twitter: number of mentions. The two major periods of activity (in red boxes) correspond to when we were following many others and during the conference, respectively.

The number of mentions over time was also observed (see Figure 8). As pointed out by Cha et al [1], mentions is a measure for the value of a name. Because our conference Twitter account @eHealthConf did not have a long history, we did not expect too many mentions. The maximum value was indeed seven mentions on a single day (during the conference) and a few mentions over the rest of the time. Nonetheless, we observed an association between the different activities we made. There were two “dense” groups of mentions, one in the beginning and one during the conference. The former was primarily related to our activity of gaining followers by simply following many others. The latter group was unsurprisingly related to the real event of the conference itself.

##### Outreach

To analyze the outreach, we looked at the number of followers and added the users receiving retweets from them. For each retweet that someone made to a tweet of our Twitter account, we received an email notification from Twitter stating the number of users that received the retweet.

**Figure 9:**
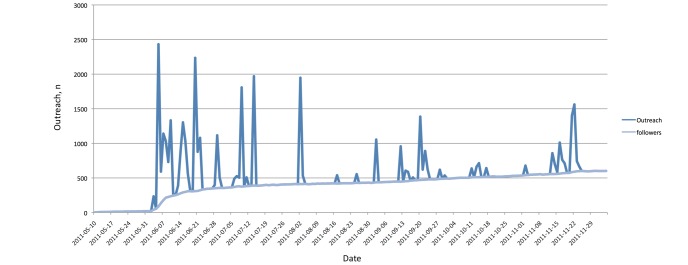
Twitter: outreach (followers plus users receiving retweets).

From Figure 9, we can observe that there are a number of high peaks. They resulted from retweets of users who had a high number of followers themselves. This meant a message from our @eHealthConf account had reached not only our followers directly, but also the followers of the user who retweeted the message. This resulted in a short-term outreach of more than 2000 users (eg, one user had more than 2400 followers and retweeted one of our messages in the early phases).

**Figure 10:**
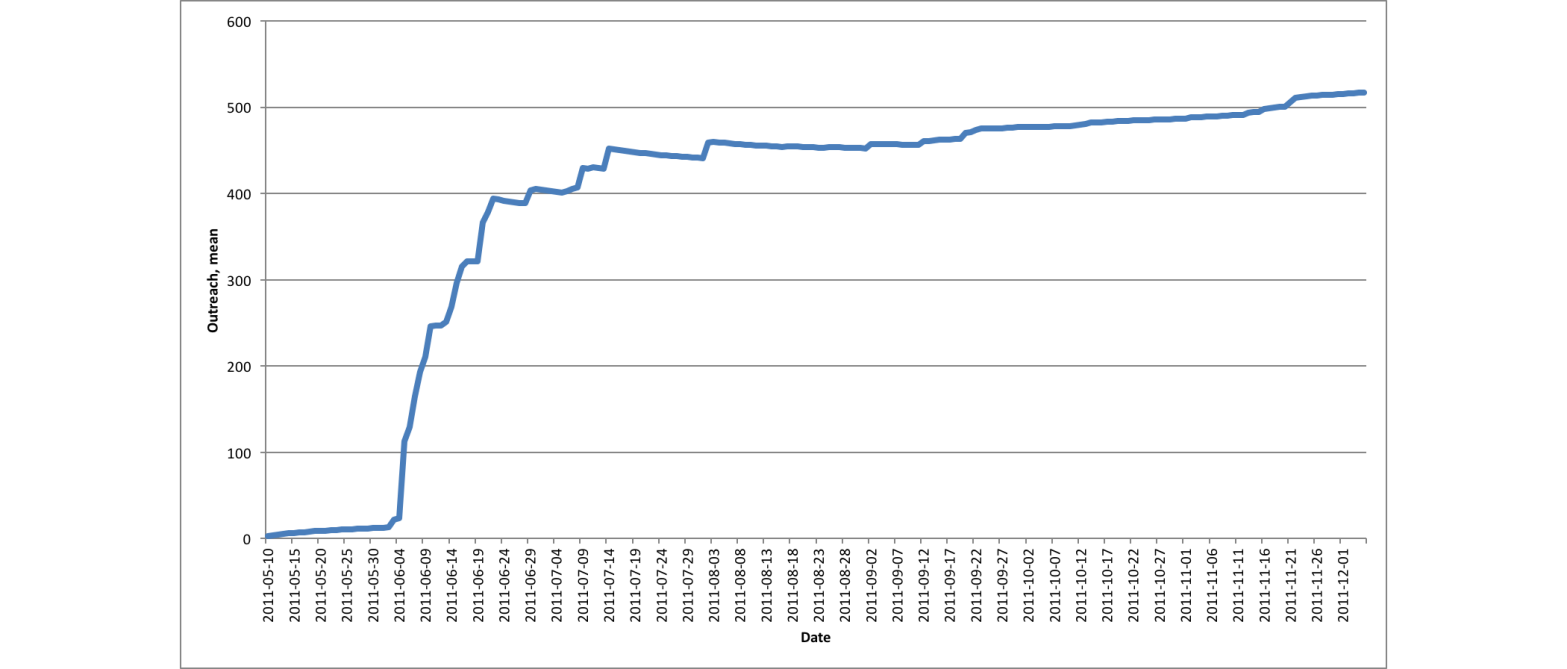
Twitter: mean outreach (arithmetic mean over time).

We also calculated the mean outreach over time. Figure 10 shows the mean outreach for the sum of our followers plus users receiving retweets. The mean outreach at a certain point in time included all other outreach values before (ie, we always calculated the arithmetic mean from day 1 to the current day).

From Figure 10, we can clearly observe the setup phase where there was only insignificant outreach. Then, once the active promotion phase started, there was first a sharp increase in the mean outreach, which later had slower growth. There was another small increase again in the last-minute promotion phase and during the actual event.

Based on the preceding numbers, we calculated the maximum and mean outreach of our Twitter account within the five different phases (see Table 2). If we compare the maximum and mean values in the different phases, we can make two obvious observations: (1) mean outreach was always higher in one phase than the previous, except for the last (postevent phase), which matched the continuous growth of followers and (2) maximum outreach had its highest value in the early promotion phase and another high value during the event (the former resulted from the retweeting of a single message by a user with a high number of followers and the latter was a combination of the increased number of followers for our own account and retweets by users with high number of followers).

In addition, we observed that in the last-minute promotion phase we had a lower maximum outreach (n=1011) compared to the earlier active promotion phase (n=2432) or the actual event phase (n=1562). However, the mean outreach was still growing in the last-minute promotion phase. Therefore, although the maximum outreach was lower, the increased mean outreach meant there was dense activity within the virtual community.

During the actual event, we had high values, both in maximum and mean outreach. Although the maximum outreach during the event (n=1562) was lower than the maximum outreach of the early promotion phase (n=2432), the mean outreach was high (mean 1236, SD 434). This resulted from dense activity during the conference in terms of tweeting and retweeting. In particular, it showed that the mean outreach could be higher when several people retweeted a message to only a few or moderate number of followers than the outreach induced by a single retweet of one user to a higher number of followers.

#### Facebook

For each of the three measures related to our Facebook page (likes, posts, and visits), we used the data on a weekly basis because they were provided by the automatic email notifications sent from Facebook. Unfortunately, this information was only gathered from the middle of July and not from the beginning of the setup phase. Nevertheless, the data showed an increase in outreach over time, with a high peak during the actual event phase similar to the outreach of our Twitter account.

##### Likes

**Figure 11:**
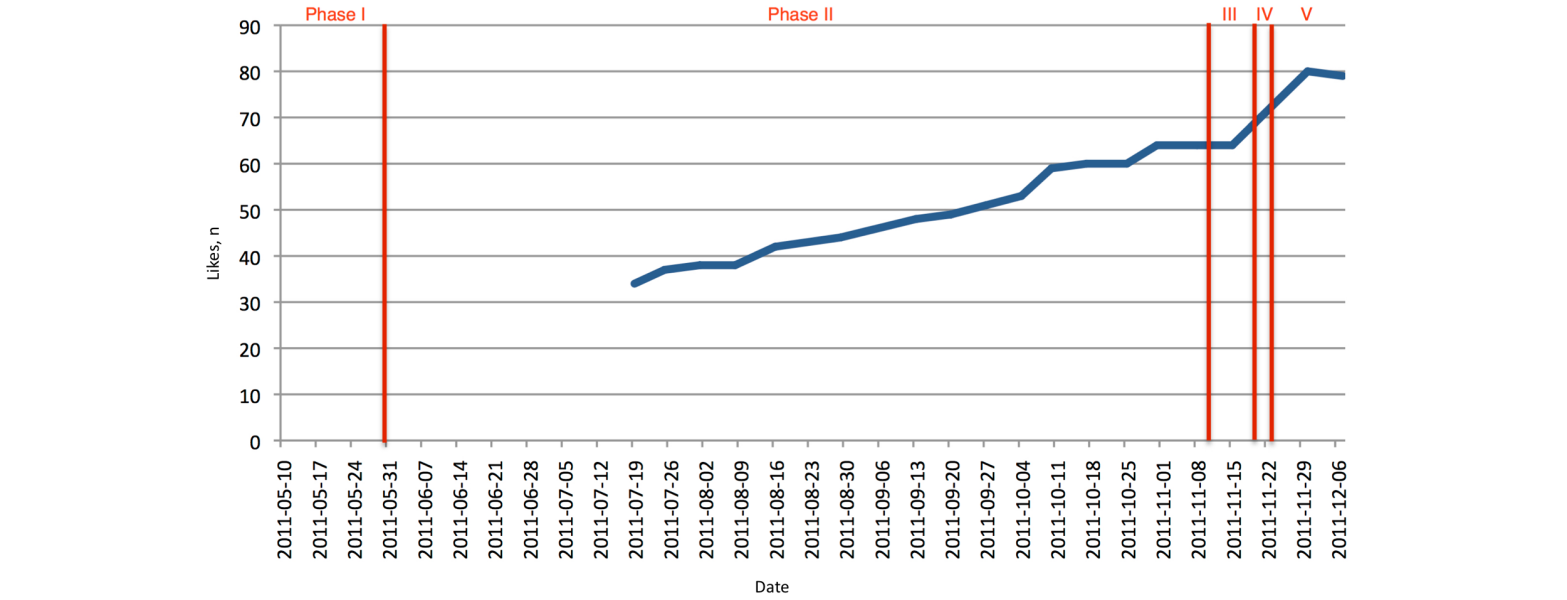
Facebook: number of likes. Note: information was only gathered from mid-July onward. Phase I: set up; phase II: active promotion; phase III: last-minute promotion; phase IV: actual event; phase V: postevent.

The number of Facebook users that “liked” our Facebook page continuously grew from only a handful (actually the Facebook user accounts of the event organizers) up to approximately 80 at the time of the conference. The growth was almost linear as Figure 11 shows.

##### Posts

**Figure 12:**
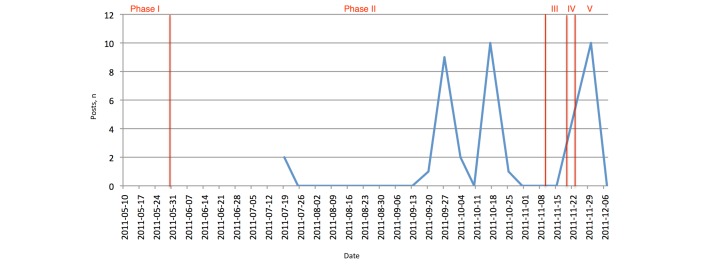
Facebook: number of posts. Note: information was only gathered from mid-July onward. Phase I: set up; phase II: active promotion; phase III: last-minute promotion; phase IV: actual event; phase V: postevent.

We counted both posts made by us on the page and also posts and comments by other users as “posts.” This measure was already included in the weekly email notifications we received from Facebook. We observed three peaks in posts/comments (see Figure 12). The first two were in September and October; this is when we posted information about the deadlines, announced invited speakers, and posted reminders about registration and the conference program. The third peak occurred around the event itself, which included information about the invited speakers and updates on the conference program.

##### Visits

**Figure 13:**
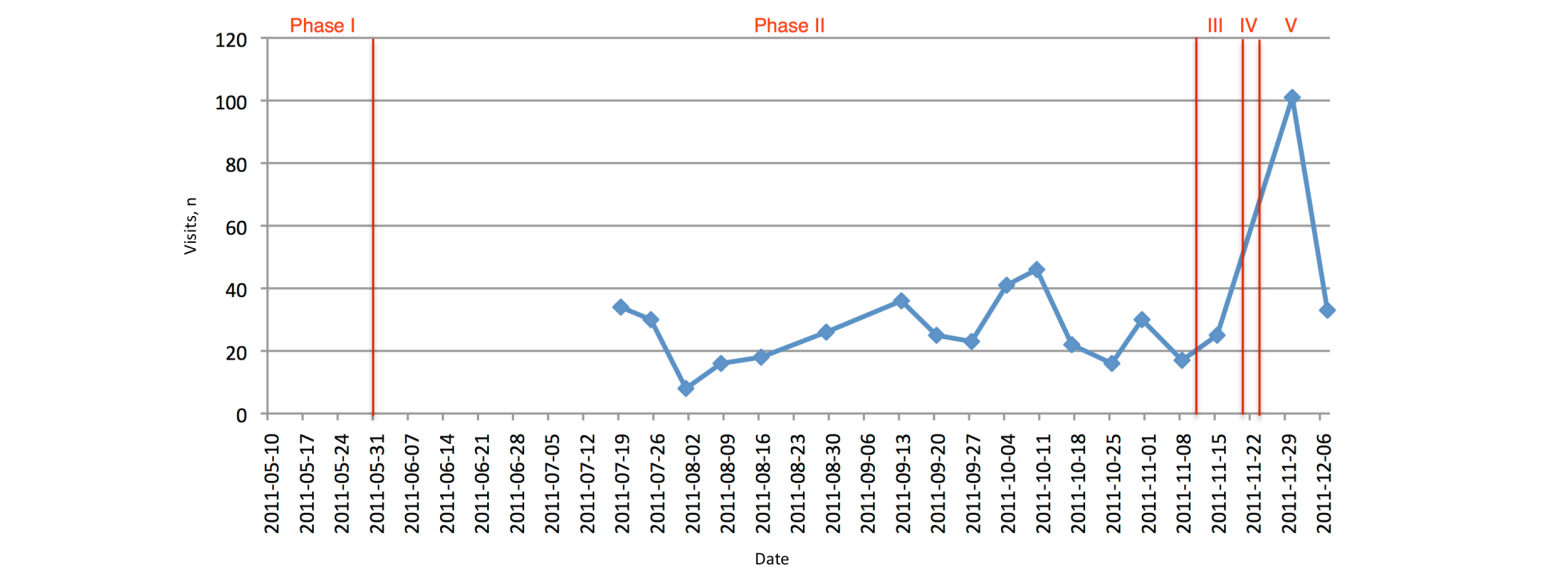
Facebook: number of visits. Note: information was only gathered from mid-July onward. Phase I: set up; phase II: active promotion; phase III: last-minute promotion; phase IV: actual event; phase V: postevent.

The Facebook weekly statistical notifications also included information about the actual visits to the page. These numbers reflected the number of people who actually looked at the page (ie, by following a post that appeared in their “news” timeline, by loading the page specifically, or by following an external link such as our Facebook-Twitter link). Figure 13 shows that there were a number of smaller peaks during the promotion phase and during the time the event took place.

##### Outreach

A measure of outreach via Facebook was the sum of the likes and the visits. These values reflected the actual readers (visits) and the potential users (likes) that we could reach in each phase. Unfortunately, we did not have a complete dataset, but only a weekly update. Hence, the outreach was determined on a weekly basis, which resulted in the same values for maximum and mean outreach for the previous three phases (see Table 2). Moreover, as the datasets started with mid-June, our outreach analysis also missed the values for the setup phase. Nevertheless, the results showed that we had increasing outreach up to the time of the actual event, with maximum and mean outreach both at 181 during the conference.

#### Website

**Figure 14:**
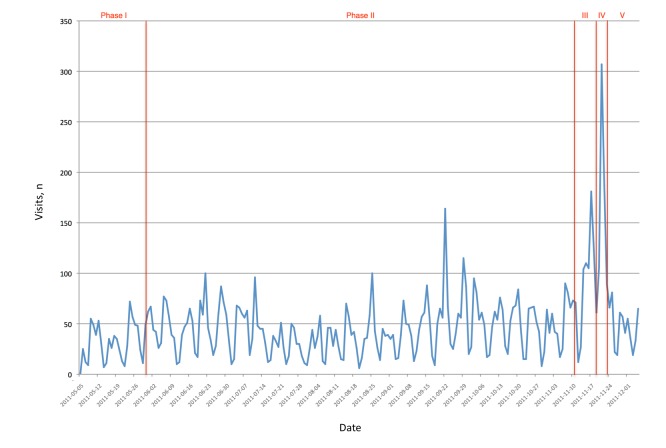
Website: number of visits. Phase I: set up; phase II: active promotion; phase III: last-minute promotion; phase IV: actual event; phase V: postevent.

Information about our conference was also available on the main conference website. This was the main information site and accounts on Twitter and Facebook always contained links to the website. The website was also the only media used to submit papers and to register for the conference. We logged the daily page visits on our website during the same time period as analyzed previously (ie, from May 2011 until begin of December 2011). Figure 14 shows the daily page visits (for the entire website) during that period.

We observed a number of high peaks (more than 100 page visits per day) on the following dates:

June 22, 2011: slightly increased number of tweets on that day and the previous two days announcing membership of senior technical program committee and selective topics from call-for-papers (total: nine tweets on June 20, 21, and 22).

August 25, 2011: three tweets on that day (venue confirmed and link to page on website, registration open and link to page on website, announcement of extension of the poster/demo submission deadline).

September 22, 2011: slightly increased number of tweets on that day and the previous two days announcing confirmed keynote speakers and link to page on website.

September 29, 2011: no clear potential cause from Twitter (there were three tweets on the day before, but they were only retweets of news from other and no link to our website).

November 14-18, 2011 (just before the conference): probably people wanted to check the latest news/changes to the conference (eg, detailed program, when the conference starts, where the hotel venue was).

November 20-23, 2011 (during the conference): assume this is primarily due to the live blogging of the conference talks from an analysis of the geographic locations of the origins of these accesses (discussed subsequently).

The preceding explanations are only potential reasons for the high peaks in the website visits because we cannot make direct correlations due to missing tracking capabilities.

**Figure 15:**
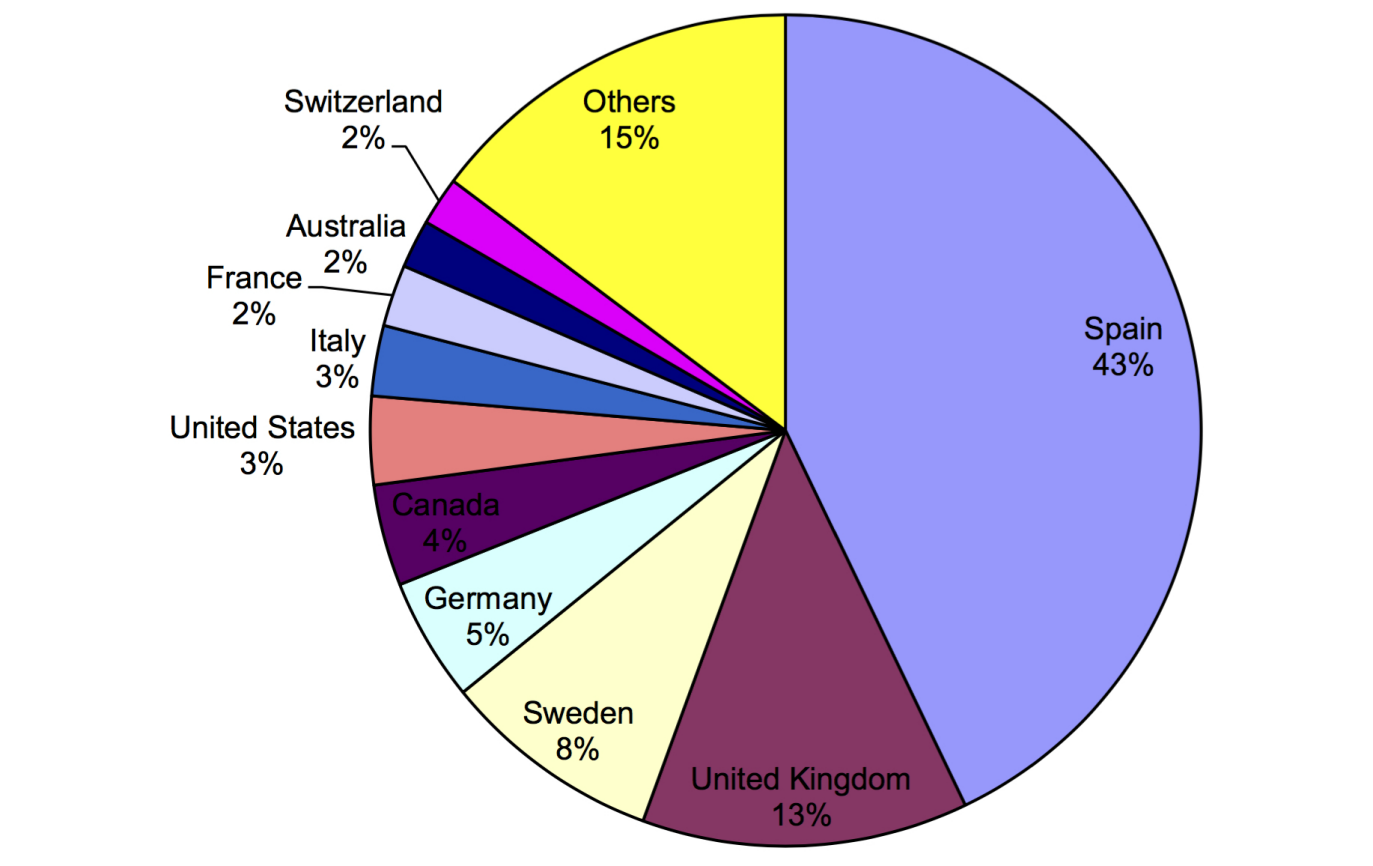
Website: visits by location.

Using Google Analytics, we found the geographical locations of visitors to the website. During the conference, there were accesses from 44 different countries (see Figure 15), which compared favorably with the number of countries represented by the conference delegates (24 different countries) and seemed to indicate that the website had a higher outreach than the physical attendance at the conference (of course, delegates physically attending the conference would receive much more information and individual benefit than those viewing the website, so this is a measure of the geographical outreach rather than the absolute impact). There were a large number of accesses from Spain; by using Google Analytics, we saw that 79% (198/250) of these were from Malaga. It seems most likely that the majority of these were from delegates in the conference venue.

#### Flickr

Poster presentations at conferences are a long-standing academic staple. Their popularity has increased in the scientific community due to their ability to quickly and efficiently communicate research, and a number of guides outlining what makes a good poster and a good poster session have been proposed [25,26]. Their general success in disseminating research activity has been widely reported (eg, [27]) but, unlike a conference paper, their impact within the academic community is time limited because they tend not to have a life outside of the conference or after the conference has finished. Although abstracts are often published to accompany the poster session, a great deal of information that is contained within the poster is often lost. Therefore, a possible solution to this problem is to make the posters themselves available to delegates, perhaps in printed form or electronically on a USB memory stick or a CD-ROM, but again this is restricted to conference delegates, many of whom will have already had the opportunity to attend the poster session.

During the previous edition of the conference (eHealth 2010), one of the authors presented a poster at the conference and also uploaded it to the popular photo sharing website Flickr along with the abstract in the description. At the time of writing, this poster has now had more than 8000 views in approximately 500 days (a mean of approximately 16 views per day). In comparison with the number of attendees at the conference poster session in 2010 who saw the poster, this is a significant increase and presents a potential method for increasing the number of views of posters and, therefore, increasing the impact and outreach of the research they represent.

##### Method

Following the success of the poster described previously, it was decided by the organizing committee of eHealth 2011 that in addition to the traditional poster session at the conference, authors would be asked to participate in an online poster session. Abstracts for the posters went through the usual peer-review process and the authors of accepted abstracts were sent instructions to upload a version of their completed poster before the conference to Flickr along with the abstract in the description and add it to a public eHealth 2011 group created by the poster chair. Six of the 10 posters were successfully added to the group, but problems were reported when attempts were made to add the posters of one of the authors. Flickr had a policy regarding recently created accounts adding photos to groups (to protect against spamming) and three posters that had been uploaded to Flickr could not be added to the group. One of the key objectives of adding photos to a group was to allow the conference organizers to provide a single link to the posters on the conference websites and related promotional activities. However, using groups in this manner was not suitable, so a workaround was found in the form of galleries. Galleries on Flickr are “a way to curate up to 18 public photos or videos of your fellow members into one place” [28]; therefore, the poster chair created an eHealth 2011 gallery and added the nine uploaded posters to the gallery and it was this link that was then promoted.

##### Results

Unfortunately, Flickr only allows users to view the distribution of page views over time within a rolling 28-day period. Therefore, the results presented here are limited to total page views only. The eHealth 2011 gallery front page was set up on November 20, 2011, during the actual event phase, with nine posters and received 520 views. However, individual posters received more page views indicating referrals from other sources (eg, search engines with indexed keywords in the poster title and description). Table 3 shows the number of page views for each poster.

**Table 3:** Number of page views on Flickr for each poster.

Poster title	Page views, n
The Guidance for Review and Approval of the U-health Care Medical Device	51
Enhancement of Sensitivity with Gathering Internet-Based Systems for Early Threat Detection Within the Global Health Security Initiative (GHSI): The EAR Project	43
Social Networks and Medical Doctors and Students	833
Epidemic Intelligence (EI) in France: Social Networking Emphasising the Process	51
A Remote Elderly Assisted Living (REAL) System	121
Representing and Accessing Scientific Knowledge About the Alzheimer’s Disease: The Semantic BiblioDem Portal	55
Review of Evaluation Processes of Web-based Systems Mining Medical Information Applied to Epidemic Intelligence	36
Reinforcing Antimicrobial Pharmacology Knowledge of Health Science Students Through a Tower Defense Video Game	138
Connect and Share: Helping Seniors with Social Isolation Use Facebook	161
Total	1489

Since November 2011, during the postevent phase, the mean number of page views per poster was 165, but a single poster received the majority of the page views (n=833). Due to not having access to the referral data for the posters, it is hard to determine why this poster received more page views than the others combined. A potential explanation is that the user may have already had a following on Flickr, but this was the only upload that this user made. More likely explanations are that the author had an active Twitter account with more than 300 followers where a link to the poster was posted and also the keyword-friendly title of the poster (“Social Networks and Medical Doctors and Students”) may have drove traffic via search engine referrals.

##### Conclusions

In total, the online poster session created more than 2000 page views to the gallery and posters. In comparison to high-traffic sites, this figure could be seen as insignificant, but there were approximately 30 delegates present at the eHealth poster session and the online poster presentation represented a percentage increase in views of approximately 6500%. In addition to page views, an online poster session increased the lifetime and permanency of the posters and also had the potential to promote discussion during the conference and after the conference ended (although no comments were made at the time of writing on any of the individual poster pages).

#### Liveblog

To make the conference more exciting for virtual participants, we ran a live blogging service by a dedicated reporter (science journalist) who attended the event specifically in this capacity. The live blogging platform CoverItLive [29] was used during the conference to implement the Liveblog. This allowed the reporter to provide live coverage during the conference so that an external audience could follow the proceedings, comment, and question the participants. The reporter also acted as an online moderator and could, if it proved necessary, block unsuitable comments and spam from being published. Very few external users commented via CoverItLive. Only three comments were made in this way and, of these, only two of these were published after moderation.

Another benefit of CoverItLive was that it also aggregated Twitter messages using the conference hashtag, which was by far the most common route for participators to make comments. On analysis of CoverItLive’s statistics, there were 416 readers of the live blog, although information on their locations was not available. At the end of the conference, the social media aspects of the conference were archived by allowing a replay of the Liveblog on the conference website.

**Table 4:** Summary of information gathered from the CoverItLive blog.

Information category		n
**Reader information**		
	Total readers	416
	Email reminders set	0
**Published entries**		
	Reporter comment	180
	Twitter comment	375
**Reader comment**		
	Reader comments sent	3
	Reader comments published	2
**Media count**		
	Images shown	1
	Newsflashes	1
**Google Analytic**		
	Number of replays	1507
Highest number of participants noted on blog at any one time (Tuesday morning)		20

Comments made by the readers were positive and ranged from an acknowledgment of being able to access and follow the conference (eg, “Learning a lot from this; thanks guys”) to more specific evaluations and questions (eg, “Ruth Hunter gave a great talk on the novel systems for behavior change...would be great to learn more about what motivates different age cohorts”).

##### Conclusion

The CoverItLive blog increased the reach of the conference to a wider audience with external participants logging in to make a connection with the conference output. The provision of the blog was effective in creating a wider and engaged audience, which allowed the conference to have a greater impact. The direct questioning of some of the speakers at the conference by the external participants demonstrated a physical community coming together virtually to take part in a real-time conference event. This suggests a future model for widening participation and the impact of scientific conferences.

Despite the lack of information about the location of the audience, it was clear that many people found the information provided useful as evidenced by the high number of views that could be seen by examining the Google Analytics information.

#### Email Lists

To further promote the event, we used existing Yahoo! email lists with a combined total of approximately 300 users to send the call-for-papers and other conference announcements. In addition, we used our own list of nearly 2000 email addresses of participants at past conferences and other events. The numbers of email addresses on the latter list at the beginning and end of the various phases are shown in Table 5. Most of the email gathering activity occurred during the setup phase. Furthermore, we estimated that approximately 5% to 10% of the emails that were sent bounced. Note that the maximum, mean, and total outreach results in Table 2 include the numbers of all email lists combined.

**Table 5:** Number of email recipients in our own list.

Phase	Email addresses at phase start, n	Email addresses at phase end, n
Setup	652	1828
Active promotion	1828	1888
Last-minute promotion	1888	1889
Actual event	1889	1889
Postevent	1889	1889

## Discussion

Our results show that the outreach of a scientific conference can be much higher than measured by traditional impact measures (ie, conference attendees and published proceedings). Despite this physical community during and after the conference, we also took online channels and social media into account. Although many scientific conferences today already use email lists and websites to promote their event, our case study shows that additional outreach can be achieved through social media. Based on the presented data, we can observe that the impact of the different types of channels varies:

Flickr: perhaps most effective (postevent) is the use of a photo gallery service such as Flickr to build a permanent “virtual poster session.” Essentially, we were able to increase the outreach of normal poster session attendees of approximately 30 people to approximately 2000 views on the Flickr gallery.

Liveblog: the use of the hashtag on Twitter and its integration into the live blogging service operated during the conference brought in more interactivity than with physical attendees only. Thus, additional questions were raised from the virtual community that were not present at the conference and members of both the virtual and physical communities discussed the actual conference presentations online.

Twitter/Facebook: Twitter was best in terms of creating a longitudinal complex stream of information (600 followers receiving regular updates vs 80 likes on Facebook). It seems that the scientific community (at least for this conference) prefers Twitter over Facebook to be informed about and discuss conferences. The popularity of Twitter might be that it is a public medium as opposed to the closed network that Facebook supports. Moreover, at the time of the conference there was no equivalent of the hashtag on Facebook. However, we cannot make a general statement here as we only measured the data for one event. In addition, we have to say that approximately 400 of the 600 Twitter followers might be a result of “follow back” behavior (ie, following other users once they are followed by them). Nevertheless, we can assume the remaining 200 followed the account driven by their own interest.

In summary, our results show that the use of Twitter and email are most effective in terms of outreach in the phases before and during the event. However, the website accumulated the highest total in the active promotion phase and Liveblog, Facebook, and the website gained more attraction during the event. For the postevent phase, although there was still some activity on Twitter, the “natural winners” are those channels that have archiving characteristics (ie, Flickr for the poster session and the offline and online proceedings). Based on these results, it could be advantageous to focus activities on these channels according to our phases.

### Limitations

This study also has a number of limitations. Most importantly, we analyzed each channel individually on its own. We did not try to identify or analyze interconnections between the various channels. For instance, it would be interesting to see how many tweets brought users to the website and the other way round, and how many clicks to the Twitter box from the home page resulted in a retweet. There might also be duplicates in terms of the actual people behind the different media users. This is why we can only speculate about the actual outreach. A more complete analysis would try to remove duplicates and identify the links between the different channels. However, some links may not be detectable (eg, anonymous webpage visitors cannot be tracked to their potentially existing social media accounts).

In addition, for the postevent phase, we only took data from a 12-day period after the conference. Of course, increased outreach could be achieved if measured for a longer period. For instance, proceedings have actually been printed and distributed a couple of months after the event. Moreover, individual paper downloads usually occur a long time after the event. Although access to the poster gallery on Flickr continues, the paper downloads are a traditional means of measuring outreach and will most likely not influence the “new” means of outreach. However, maintaining a virtual community for an extended period after a real event might be useful and important (eg, to support the next event related to the previous one because, typically, scientific conferences happen annually).

One final limitation of this study is that factors relating to effort and cost-effectiveness have not been considered. There is clearly the potential to reduce effort in some of the activities (eg, by linking social media accounts so that a single post appears on various channels), but there were considerable costs for the authors with respect to time taken to create the accounts, formulate strategies, and then create and post content. For practical use, these factors need to be carefully identified and further work is needed in this area to gauge whether the use of freely available channels such as Twitter are cost-effective in comparison to more static channels, such as the website, which have considerable setup costs.

### Future Work

Future work could try to find the interconnectivity in the outreach of the different media channels and the link between the virtual community growth and the real community behind it. For some channels, more intensive data crawling could help to find these analyses (eg, linking the Twitter users that received retweets to previous retweets or other involvement). Other findings might only be possible by tracking known users or asking them for their consent to reveal their online identity and use this data (eg, linking a user on Twitter to a real person who might be an attendee at the conference and a visitor of the website).

Further research could aim to generate a social network and understand the topological changes caused by such an event (eg, what is the rate of the increased density of the social network as a result of meeting in person at a conference?). A possible way to collect a richer dataset for this purpose would be to start the usage of Twitter earlier and build up a larger number of followers. For example, the USENIX Association has a Twitter account (@usenix) with more than 4100 followers (at the time of writing this paper), which has existed since November 2008. This account is reused to announce and promote various conferences organized by USENIX. An important factor here is to maintain the community for a series of events. Applying our approach to accounts and organizations such as these would allow for even larger longitudinal analyses.

Finally, future work could add live stream audio/video from the conference to the website to better engage with virtual participants. Unfortunately, this was not possible at eHealth 2011 for local logistical reasons. Other conference series use this type of media already to increase their outreach (eg, the Chaos Communication Congress and the USENIX Annual Technical Conference provide audio and video records of their events on their website). However, the effects of this outreach and its meaning compared to other media channels have not been analyzed in a longitudinal fashion yet.

### Conclusion

In this paper, we present a robust framework to define a physical and virtual community around an event and the role and effectiveness of online and social media usage in the promotion and presentation of a scientific conference.

The main approach is to establish a virtual community around the physical community of the real event; we also established five phases for event promotion (setup, active promotion, last-minute promotion, actual event, postevent) with the aim to observe the community growth behavior over the five phases around the event. In contrast to existing works, we made a comparative analysis of the media channels and a longitudinal study rather than looking at snapshots of data from a single medium. We also combined a virtual and a physical community, analyzed their growth and behavior over the five phases with respect to dissemination of scientific outputs and outreach, and we measured the outreach and engagement by two-way communication (ie, we were promoting the event and mining the data about the promotion at the same time). To illustrate our approach, we presented a case study of a real scientific event, the eHealth 2011 conference, which took place in Malaga, Spain, in November 2011. As we ran the conference, we also had a unique opportunity to develop and measure the outreach strategy of the conference with full understanding and insight into the social media strategy rather than just analyzing social media data of a random event.

The main achievement was the novel generalizable framework and we found insights into one conference outreach using our novel method. Our framework includes five phases for event promotion (setup, active promotion, last-minute promotion, actual event, postevent), defines virtual and physical communities, defines outreach and impact measures, and provides guidelines to measure the outreach of separate social media channels. Results from our case study of the eHealth 2011 conference revealed that it seems advantageous to focus on different media channels in each of the five phases: A mix of Twitter, email, and a website can be recommended to achieve the highest outreach before the conference, and these channels can be extended with Facebook and a Liveblog during the event, whereas the best channels after the event were Twitter and (for the long run) Flickr and proceedings. This is a cornerstone of research into a more robust understanding and analysis of social media promotion strategies for conference organizers who wish to apply our framework.

## Appendix

Multimedia Appendix 1 Comparison of traditional and new impact measures.

Multimedia Appendix 2 Media channels of the eHealth 2011 conference.

Multimedia Appendix 3 Promotion timeline of the eHealth 2011 conference.

## Abbreviations

API: application programming interface
RFID: radio-frequency identification
