# Supplementary material for: Follow #eHealth2011: Measuring the Role and Effectiveness of Online and Social Media in Increasing the Outreach of a Scientific Conference
Source: J Med Internet Res. 2016 Jul 19;18(7):e191. doi: 10.2196/jmir.4480 (PMC4971392; doi:10.2196/jmir.4480)
Supplement: Multimedia Appendix 1 [file jmir_v18i7e191_app1.pdf]

Multimedia Appendix

1. Comparison of traditional and new impact measures

<table>
<tr><th>Traditional impact measures</th><th>New impact measures</th></tr>
<tr><td>E-Mail: number of emails sent/forwarded</td><td>Twitter: official conference account
• number of followers
• no. of retweets
• no. of favourites
• no. of users receiving retweets
• no. of mentions of account name</td></tr>
<tr><td rowspan="2">Proceedings: number of:
• citations of papers
• downloads of papers
• sold printed copies</td><td>Twitter: external engagement – hashtag use:
• number of followers of any user who mentions the hashtag
• no. of retweets
• no. of mentions of hashtags
• no. of favourites
• no. of users receiving retweets</td></tr>
<tr><td>Flickr: number of views of posters</td></tr>
<tr><td>Conference: number of participants</td><td>Liveblog: number of
• people who posted questions from outside of the event
• real time online views</td></tr>
<tr><td>Website: number of page visits</td><td>Facebook: number of page visits, likes, posts</td></tr>
</table>
