# Supplementary material for: Follow #eHealth2011: Measuring the Role and Effectiveness of Online and Social Media in Increasing the Outreach of a Scientific Conference
Source: J Med Internet Res. 2016 Jul 19;18(7):e191. doi: 10.2196/jmir.4480 (PMC4971392; doi:10.2196/jmir.4480)
Supplement: Multimedia Appendix 2 [file jmir_v18i7e191_app2.pdf]

Multimedia Appendix

2. Media channels of the eHealth 2011 conference

Channel	Instantiation
Twitter	http://twitter.com/eHealthConf; Hashtag: #ehealth2011
Facebook	http://www.facebook.com/eHealthConf
Flickr	https://www.flickr.com/photos/eddequincey/galleries/72157628028815537/
Liveblog	http://www.coveritlive.com (embedded into the Website)
E-Mail	Yahoo! email lists (self maintained)
Website	http://electronic-health.org/
