# Supplementary material for: Follow #eHealth2011: Measuring the Role and Effectiveness of Online and Social Media in Increasing the Outreach of a Scientific Conference
Source: J Med Internet Res. 2016 Jul 19;18(7):e191. doi: 10.2196/jmir.4480 (PMC4971392; doi:10.2196/jmir.4480)
Supplement: Multimedia Appendix 3 [file jmir_v18i7e191_app3.pdf]

Multimedia Appendix

3. Promotion timeline of the eHealth 2011 conference

	Date	Activity
**Phase**		
Setup	10 May<br><br><br>12 May<br><br>20 May<br>27 May	Call-for-Papers (CFP) sent to email lists; set up Twitter account and Face- book page; posted CFP and announced official Twitter hashtag<br>Started to post news messages related to conference's topics on Twitter and Facebook<br>Twitter stream widget added to official website<br>2nd CFP sent to email lists
Active Promotion	1 June<br>6 June<br><br><br>21 June<br>28 June<br>13 July<br><br>19 July<br><br><br><br>2 Aug<br><br>25 Aug<br><br>14 Sep<br><br>28 Sep<br><br><br>29 Sep<br><br>2 Nov	Started to follow people on Twitter<br>Started Twitter promotion (2 tweets per day asking for submissions)<br>Announced CFP deadline on Twitter and by email<br>Announced extended CFP deadline on Twitter and email<br>Sporadically tweeting and retweeting ehealth-related news (during the week)<br>From this date tweets were mainly focussed on conference news (reviewing completed, program online, etc.); retweeting of other news messages was stopped/reduced<br>Started to announce submission deadline for posters and demos<br>Announced deadline extension for posters and demos<br>Registration was opened, venue was confirmed<br>Started to ask on Twitter for questions to be discussed at the conference panel<br>Decided for now to stop asking for questions for the panel due to lack of response, and to ask again nearer to the time of the conference<br>Created the Flickr group (later a Flickr gallery) for poster authors to upload images<br>Sent reminders to register for conference, sporadically retweeting ehealth- related news
Last Minute Promotion	13 Nov<br>15 Nov<br>16–17 Nov	Announced the lineup of invited speakers<br>Created the live blog<br>Asked again on Twitter for panel questions; announced live blog
Actual Event	21–23 Nov	Conference (live blogging / tweeting)
Post Event	24 Nov – 5 Dec	Post event communication
